# Anti-biofilm effects and characterisation of the hydrogen peroxide activity of a range of Western Australian honeys compared to Manuka and multifloral honeys

**DOI:** 10.1038/s41598-019-54217-8

**Published:** 2019-11-27

**Authors:** Azhar Sindi, Moses Van Bawi Chawn, Magda Escorcia Hernandez, Kathryn Green, Md Khairul Islam, Cornelia Locher, Katherine Hammer

**Affiliations:** 1 School of Biomedical Sciences, The University of Western Australia, Crawley, Western Australia 6009 Australia; 2 The Cooperative Research Centre for Honey Bee Products Limited, Western Australia, Australia; 3 School of Allied Health, The University of Western Australia, Crawley, Western Australia 6009 Australia

**Keywords:** Antimicrobials, Infectious diseases, Medicinal chemistry

## Abstract

The antibacterial activity of honeys derived from the endemic flora of the southwest corner of Western Australia, including the trees Jarrah (*Eucalyptus marginata*) and Marri (*Corymbia calophylla*), remains largely unexplored. Investigation of these honeys showed minimum inhibitory concentrations (MICs) of 6.7–28.0% (w/v) against Gram positive and negative bacteria. Honey solutions showed enhanced antibacterial activity after hydrogen peroxide was allowed to accumulate prior to testing, with a mean MIC after accumulation of 14.3% compared to 17.4% before accumulation. Antibacterial activity was reduced after treatment with catalase enzyme, with a mean MIC of 29.4% with catalase compared to 15.2% without catalase. Tests investigating the role of the Gram negative outer membrane in honey susceptibility revealed increases in activity after destabilisation of the outer membrane. Honeys reduced both the formation of biofilm and the production of bacterial pigments, which are both regulated by quorum sensing. However, these reductions were closely correlated with global growth inhibition. Honey applied to existing biofilms resulted in decreased metabolic activity and minor decreases in viability. These results enhance our understanding of the mechanisms of antibacterial action of Jarrah and Marri honeys, and provide further support for the use of honey in the treatment of infected wounds.

## Introduction

Honey is a complex natural product produced by bees from the nectar of melliferous plants. Honey contains primarily sugars (approximately 74–78%), including mono-, di- and tri-saccharides, and water (about 16–18%)^1–3^. Honey also contains a relatively minor, yet highly variable and complex fraction of proteins (including enzymes)^4,5^, organic acids, vitamins, minerals and plant-derived phenolic compounds^6^. The antimicrobial activity of honey produced by the European honeybee *Apis mellifera* is well documented^7,8^, and is attributed largely to the high osmolarity, relatively low pH (around 4.5) and the production of hydrogen peroxide^9,10^ in addition to the actions of other plant^11^ or bee^12,13^ derived compounds. The antimicrobial activity of different honeys varies substantially *in vitro*, and is related to variations in the above factors, many of which are directly related to the specific floral source that the honey is derived from. Distinct differences in the levels of activity^7,14–16^ and mechanisms of antibacterial action^17^ of honeys with a hydrogen peroxide activity component to those without (for example Manuka honey), have been reported. In Manuka honey, which is derived from specific *Leptospermum* species, the chemically stable^18^ compound methylglyoxal contributes substantially to the bactericidal activity, whereas in so-called “peroxide honeys”, methylglyoxal is essentially absent and bactericidal activity is due mainly to the generation of hydrogen peroxide^17^ and the resulting generation of hydroxyl radicals^16^.

The antimicrobial activity of honey can be quantified in many different ways. Determining minimum inhibitory concentrations (MICs) of honey is relatively straightforward technically, and allows for direct comparison of results generated in different studies, as long as the methodology is sufficiently similar. For example, particularly low MICs have been reported for a number of honeys, including multifloral and Buckwheat honeys from Poland (MICs of 1.56%)^19^, heather honey from Scotland (MICs < 2%)^20^ and chestnut, fir and forest honeys from Slovenia (MICs of 2.5%)^21^. In addition, a range of MICs from 3%^22^ to 25%^23^ have been published for Manuka honey, whereas higher MICs of 32% (w/v) have been reported for Australian multifloral honeys with no specific floral source^8^. Furthermore, examples of MICs for honeys with a hydrogen peroxide component range from 8–32% for Jarrah honey^8^, 4–16% for several Canadian honeys including buckwheat^16^ and 6.25–25% for several polyfloral honeys from Greece^24^. Quantification of additional antibacterial effects allows further insight into the varying antibacterial mechanisms of honeys. Specifically, the effects of honeys on microbial biofilms and virulence are particularly relevant given that honey is reportedly an ideal treatment or dressing for chronic, non-healing wounds^25^. Many publications have described the effects of different honeys on biofilms^15,26–28^, however, few have specifically investigated the anti-biofilm effects of Western Australian honeys. Infection, and the presence of microbial biofilm may contribute substantially to the non-healing nature of chronic wounds^29,30^, and whilst the clinical effectiveness of honey for treating chronic wounds requires further investigation^31^, pre-clinical investigation of the underlying antibacterial mechanisms of honey is warranted.

The aim of this study was therefore to investigate several facets of the antibacterial activity of the two Western Australian honeys Jarrah and Marri, including both the contribution of hydrogen peroxide and effects on biofilm and virulence, and to compare activity to both multifloral and Manuka honeys.

## Methods

### Honey samples and preparation of honey solutions

Honeys from the floral sources Jarrah (*Eucalyptus marginata*) and Marri (*Corymbia calophylla*) were obtained from a local apiary in Western Australia. Samples Jarrah 1 (JAR1) and Marri 1 (MAR1) were obtained in March 2017, and samples Jarrah 2 (JAR2) and Marri 2 (MAR2) were obtained in March 2018. Floral sources for honeys were identified informally by beekeepers according to the major species flowering at the collection sites at the time of honey collection. Multifloral honey (MF; Capilano Honey Ltd., Bayswater, WA) with no specific floral source was purchased from a supermarket and Activon Medical Grade Manuka honey (MAN; Advancis Medical; Lot WO017671) was purchased from Independence Australia, Melbourne. The methylgloxal level in the Manuka honey was not stated. Artificial honey (ART) was prepared as described previously^22^ by dissolving 1.5 g sucrose, 7.5 g maltose, 40. 5 g fructose and 33.5 g glucose in 17 ml of sterile distilled water. All honeys were stored at room temperature in the dark for the study duration. For antibacterial activity studies, solutions of honey were prepared weight per volume (w/v) in sterile distilled water, then sterilised by passing through a non-sterile 0.7 μm glass fibre filter to remove larger particles, followed by a sterile 0.22 μm pore size filter. Honey solutions were used in antibacterial activity assays within 1 h of preparation unless stated otherwise.

### Determination of physicochemical parameters

Hydrogen peroxide levels were determined using o-dianisidine and horseradish peroxidase reagents as described previously^17^. In brief, each honey was prepared as a 30% (w/v) solution in sterile distilled water and incubated at room temperature (~22 °C). After 1, 2, 4, 6 and 24 h, aliquots were removed for analysis and o-dianisidine and horseradish peroxidase reagents were added^17^. The reaction was stopped after 5 min by the addition of 6 M sulphuric acid, and the absorbance was determined at 540 nm. Blanks were prepared for each honey and contained all reagents as described above, omitting the o-dianisidine and horseradish peroxidase. Hydrogen peroxide levels were determined from a standard curve prepared each time using doubling dilutions of hydrogen peroxide solution ranging from 550–2.1 µM. The pH of each honey was measured by dissolving 1 g of honey in 7.5 ml of carbon-dioxide free water^32^, then determining the pH with a calibrated pH meter. Colour was determined by dissolving each honey in sterile distilled water to 50% (w/v) then determining the optical density at both 450 nm and 720 nm^32^. The difference in the two optical density values was determined, then multiplied by 1000 and expressed in milli-absorbance units (mAU). Colour values were determined for all honeys both before and after passing through a 0.7 μm glass fibre filter. Total phenolic content was quantified colourimetrically at 760 nm after treatment with Folin-Ciocalteu reagent as described previously^33^. Total phenolic content was expressed as mg of Gallic Acid Equivalent (GAE) per 100 g of honey.

### Antibacterial activity testing

Reference isolates were obtained from the School of Biomedical Sciences at The University of Western Australia, and from PathWest Laboratory Medicine WA, and were as follows; *Staphylococcus aureus* ATCC 25923, *Staphylococcus aureus* ATCC 700699, *Enterococcus faecalis* NCTC 775, *Pseudomonas aeruginosa* ATCC BAA-47, *P. aeruginosa* ATCC 27853, *Escherichia coli* NCTC 10538 and *E. coli* ATCC 25922. All organisms were maintained on blood agar stored at 4 °C.

For the standard zone of inhibition assay, colonies from an overnight culture of *S. aureus* ATCC 700699 on blood agar were suspended in 0.85% saline, the density of the suspension was adjusted to approximately 1.5 × 10^8^ colony forming units (CFU)/mL using a nephelometer, and it was then used to swab-inoculate Mueller Hinton Agar (MHA) plates. Wells of 8 mm diameter were cut into the MHA plates and 100 μL volumes of each honey solution at 25% (w/v) were dispensed into wells. Sterile distilled water was used as a negative control and a trimethoprim disc (5 µg; Oxoid, Hampshire, UK) was used as a positive control. After incubation of plates at 37 °C for 24 h, zones of inhibition were measured. Wells with no inhibition were assigned a value of 8 mm (equal to the well size) to allow for statistical analysis. The phenol equivalence or “total activity” assay was performed as described previously^7,34^. Briefly, a standardised inoculum of *S. aureus* ATCC 25923 was added to 150 ml of molten Nutrient Agar and poured into a 245 × 245 mm square bioassay dish (Thermo Scientific Nunc NUN240835). After storage of the dish at 4 °C overnight, 8 mm wells were cut into the agar and solutions of 25% (w/v) honey were added to duplicate wells. Volumes of phenol solutions (2, 3, 4, 5, 6 and 7% w/v) in distilled water were added to wells in duplicate to generate a phenol standard curve. A trimethoprim disc (5 µg; Oxoid, Hampshire, UK) and sterile distilled water were used as controls. After incubation of the bioassay dish all zones were measured and zone sizes for each honey were expressed relative to phenol^7,34^.

Minimum inhibitory concentrations (MICs) of honey were determined using the broth microdilution method described by the Clinical and Laboratory Standards Institute^35^, with minor modifications. Briefly, inocula were prepared by culturing strains on blood agar overnight at 37 °C, then suspending colonies in 0.85% saline. Cell suspensions were adjusted to approximately 1.5 × 10^8^ CFU/mL using a nephelometer. Adjusted suspensions were then diluted 1 in 40 in 4 × Mueller Hinton Broth (MHB; Oxoid, Hampshire, UK). Quadruple strength MHB was required to compensate for subsequent dilution with honey in the microtitre plate, and to ensure a final concentration of 1 × MHB in the assay. To prepare honey dilutions, stock solutions of honey at 40% (w/v) were prepared and aliquoted in varying volumes into wells of a flat bottomed 96-well microtitre plate. Sterile distilled water was added to wells as required to make the volume in each well up to 150 µl. Next, 50 µl of inoculum (in 4 × MHB) was added to all wells to result in final inocula concentrations of approximately 5 × 10^5^ CFU/ml. Final concentrations of honey ranged in 2% increments from 0 to 30%. A well containing growth medium and inoculum, but no honey, served as the positive growth (untreated) control well. Microtitre plates were incubated for 24 h at 37 °C statically, after which MICs were determined visually as the lowest concentration of honey resulting in an optically clear well. For multifloral and artificial honeys testing was repeated with higher concentrations of honey, using initial stock solutions of 56% (w/v) honey, resulting in final test concentrations ranging from 42 to 22%, in 2% increments. Imputed values were used for any off-scale results to enable statistical analysis. For this, any MIC exceeding the maximum test concentration was assigned a value equal to the maximum test concentration plus 2%. For example, values of >30% were assigned a value of 32%. MICs of hydrogen peroxide (Sigma-Aldrich H1009) were also determined as described above, with final hydrogen peroxide concentrations after inoculation of 0, 128, 256, 512, 1024, 2048, 3072 and 4096 µM.

The MIC assay was also performed after the addition of the enzyme catalase to honey solutions. Catalase degrades hydrogen peroxide thereby allowing evaluation of the contribution of hydrogen peroxide production to antibacterial activity. The assay was performed as described above with the following modification; a solution of honey at 50% (w/v) was divided into two portions, after which a filter-sterilised solution of catalase (5600 U/mL; Sigma-Aldrich, St Louis, MO, USA), was added to one portion whilst the equivalent volume of sterile distilled water was added to the other portion, to result in final concentrations of 40% honey for both solutions. The honey solutions were then dispensed into microtitre plates, inoculated and incubated as described above. Controls containing no honey (positive growth control), and no honey with catalase (catalase only control) were included to evaluate the effect of catalase alone on bacterial growth.

The MIC assay was also performed after hydrogen peroxide was allowed to accumulate in solutions of honey at 30% w/v incubated statically for 3 h at room temperature (~22 °C). This honey concentration was selected as it was also used for determining levels of hydrogen peroxide as part of the physicochemical analysis, and therefore allows direct comparison. Final concentrations of honey for this variation to the MIC assay ranged from 0 to 22% (w/v), in 2% increments. To evaluate whether the pH of the 30% solutions of honey changed substantially over time, pH was determined at 0, 1, 2 and 3 h for each honey.

The final variation of the MIC assay was the inclusion of several membrane-modifying compounds in the test medium in addition to honey, with the aim of evaluating the extent to which bacterial membrane properties contribute to honey susceptibility. The test organisms were *P. aeruginosa* ATCC BAA-47 and ATCC 27853 and *E. coli* NCTC 10538 and ATCC 25922. Polymyxin B nonapeptide (PMBN; Sigma-Aldrich, St Louis, MO, USA) and ethylenediaminetetraacetic acid (EDTA) were each added to modify the outer membrane, and carbonyl cyanide m-chlorophenyl hydrazone (CCCP) was added to modify the cytoplasmic membrane. The appropriate final concentration of each membrane modifying agent was determined from preliminary experiments to be 0.5 × MIC for all compounds (data not shown). Novobiocin (256 to 8 μg/mL) was included as a positive control compound for assays with PMBN. Three honeys (Jarrah 1, Marri 1 and Manuka) were evaluated in this variation, each at final concentrations ranging from 0% to 25% (in 2.5% increments). Wells with honey, but without membrane modifiers, inoculated as above were used as “honey only” comparators. Positive (untreated) growth control wells contained inoculum and growth medium, with no membrane modifier and no honey. Controls containing no honey but with each membrane modifier added were also included to evaluate the effect of each compound alone on bacterial growth.

### Biofilm formation and quantification assay

Biofilm formation and quantification was conducted in flat bottomed 96-well microtitre plates, based on previously described methods^36,37^. To prepare inocula, *S. aureus* ATCC 700699, *E. faecalis* NCTC 775 and *P. aeruginosa* ATCC BAA-47 were grown overnight in 5 mL of Trypticase Soy broth at 37 °C with shaking. Overnight cultures were adjusted to approximately 10^8^ CFU/mL in 0.85% saline using a nephelometer, then diluted 1 in 100 in 4 × Bolton Broth to obtain approximately 10^6^ CFU/mL. Bolton broth (BB) was used as the biofilm formation medium to ensure consistency across all biofilm experiments, including the multispecies biofilm model^38^ described below. Preliminary experiments showed that quantities of biofilm formed in BB were similar to those formed in Trypticase Soy broth (data not shown). The effect of honey on the formation of biofilm was evaluated by culturing each organism in BB in the presence of several different concentrations of each honey. To achieve this, solutions of honey were prepared at 40% (w/v) and were aliquoted in appropriate volumes into wells of a 96-well microtitre plate (Nunclon delta surface microtitre plate Nunc 167008), after which appropriate volumes of sterile distilled water were added as diluent. After the addition of 50 µl of inoculum in 4 × BB, final honey concentrations were 0, 5, 10, 20 and 30% in final volumes per well of 200 µl. Triplicate wells were used for each honey concentration. Positive growth control wells (n = 12) were included in each microtitre plate and contained 150 µL of sterile distilled water (instead of honey) and 50 µL of inoculum. Negative control wells (n = 12) were also included, which contained sterile medium only. All microtitre plates were incubated at 37 °C for 24 h statically. The absorbance of all wells at 600 nm was determined using a microplate spectrophotometer at time zero prior to incubation, and again after 24 h incubation to quantify total bacterial growth.

After incubation, well contents were removed by inverting microtitre plates, after which all wells were rinsed gently twice with 200 µL of 0.85% saline to remove any remaining non-adherent planktonic cells. Plates were then drained in an inverted position, followed by air drying at 37 °C. Next, biofilms were fixed by adding 200 µL of methanol to each well and leaving for 15 min. The methanol was removed by inverting plates, followed by air drying. Then, 200 µL of Hucker’s crystal violet was added to each well for 15 min to stain the biofilm. Excess stain was removed by rinsing each plate under gently running tap water and plates were then air dried. Finally, the remaining stain was eluted by adding 200 µL of 33% (v/v) glacial acetic acid to each well and leaving for 15 min. The absorbance of all wells was then determined at 570 nm after 10 s of linear shaking within the microplate spectrophotometer. The absorbance of the corresponding blank wells (media only) was subtracted from the absorbance of biofilm-containing wells. The average of replicate wells was then determined per experiment.

### Effects of honey on formed biofilms

To form biofilms, inocula of *S. aureus* ATCC 700699, *E. faecalis* NCTC 775 and *P. aeruginosa* ATCC BAA-47 were prepared and standardised to final concentrations of 5 × 10^6^ CFU/ml in BB as described above. Volumes of 200 µl were dispensed into wells of a flat bottomed 96-well microtitre plate and plates were then incubated statically at 37 °C for 24 h to allow the biofilms to form. The optical density at 600 nm was determined at time zero prior to incubation and again following the 24 h incubation to enable the evaluation of microbial growth. After the 24 h biofilm formation period, well contents were removed by inverting plates as described above. Next, 200 µL of each honey at 20% (w/v) and 50% (w/v) in sterile distilled water was applied to the biofilms, which were then incubated statically at 37 °C for 24 h. Untreated control wells contained BB in place of honey solution. Four replicate wells were used per treatment or control. After incubation the metabolic activity of biofilms was quantified using the metabolic indicator compound 2,3,5-triphenyltetrazolium chloride (TTC) as described previously^39^ with minor modifications. Before the addition of the TTC indicator, all well contents were removed by inversion and wells were then rinsed gently once with 0.85% saline. This step was necessary to prevent interference between the honey and TTC, as preliminary studies indicated that the formation of the coloured product triphenyl formazan was inhibited in the presence of honey. A 200 µL volume of BB and 50 µL of 0.1% TTC was then added to each well, resulting in a final concentration of 0.02% TTC. The optical density of all wells was immediately determined at 540 nm to serve as the assay blank. Microtitre plates were then incubated statically in the dark at 37 °C for 2 h, after which the optical density was again determined at 540 nm. Blank values were subtracted from final optical density values and averages of replicate wells were determined. Development of a pink to red colour indicated metabolic activity.

The Lubbock Chronic Wound Pathogenic Biofilm (LCWPB) model^38^ was used to evaluate the effect of honey on multispecies biofilm within a simulated chronic wound environment. To prepare inocula, overnight cultures of the three organisms were suspended in 0.85% saline and adjusted to approximately 10^8^ CFU/ml as described above, and equal volumes of each were added to 0.85% saline to result in a multispecies inoculum containing approximately 10^6^ CFU/ml of each organism. To simulate the chronic wound environment, a medium containing 5% horse red blood cells (RBCs), 50% horse plasma and 1 × BB was prepared^38^. Each 1 mL of LCWPB media contained the following components: 500 µL plasma, 112.5 µL 4 × BB, 100 µL RBCs and 287.5 µL of sterile distilled water. LCWPB medium was dispensed in 1 ml volumes into glass test tubes (17 × 61 mm) with screw caps, which also contained a sterile plastic pipette tip (200 µL size), which provided a surface for biofilm formation. Tubes were then inoculated with 10 µl of the mixed inoculum and incubated at 37 °C with shaking for 24 h to enable biofilm formation. The formation of biofilm was confirmed by Gram staining, which showed the presence of both Gram positive and Gram negative bacteria, and by Alcian blue staining^40^, which confirmed the presence of extracellular material (data not shown).

Multispecies biofilms were then treated with honey by adding 5 ml volumes of 60% (w/v) honey prepared in sterile distilled water, resulting in a final honey concentration of 50%. A 5 ml volume of 0.85% saline was added to the untreated control instead of honey. Tubes were not sonicated before or after the addition of honey in order to more closely resemble a wound scenario. Biofilms with honey were incubated for 24 h at 37 °C with shaking, after which time viable counts were performed. Biofilms were sonicated briefly^41^ to break up clots and dislodge bacterial cells prior to viable counting. To achieve this, tubes were sonicated for 2 min (Branson 1510 ultrasonic cleaner) followed by 2 min of vortex mixing. Sonicator settings included an ambient temperature (~22 °C) and a frequency of 40 kHz. Sonicated biofilm solutions were immediately serially diluted 10-fold in 0.85% saline and 100 µl volumes were then spread onto solid media for colony counts. MacConkey agar was used to detect *P. aeruginosa* and plates were incubated at 37 °C for 24 h. Mannitol Salt Agar (MSA) was used to detect *E. faecalis* and *S. aureus*, and plates were incubated at 37 °C for 48 h. Colonies of *S. aureus* and *E. faecalis* were differentiated on the basis of colony morphology. Viable count data were log_10_ transformed and the mean and standard deviation determined.

### Quantification of the bacterial pigments violacein and pyocyanin

The two pigment-producing strains *Chromobacterium violaceum* ATCC 12472 and *P. aeruginosa* ATCC 27853 were each cultured with several concentrations of honey, then total bacterial growth was estimated by optical density, and pigments were extracted and quantified. Inocula were prepared by culturing organisms overnight in Luria Bertani (for *C. violaceum*) or Nutrient (for *P. aeruginosa*) broth at 37 °C statically, then adjusting each culture by diluting in 0.85% saline to approximately 2 × 10^8^ CFU/ml using a nephelometer. A 50 µl volume of inocula was then added to each well of a 24-well plate containing growth medium and final concentrations of 2.5, 5, 10 and 15% (w/v) of each honey, with a final well volume of 2.05 ml. The untreated control consisted of growth medium and inoculum only. For pigment production assays, *C. violaceum* was cultured in Luria Bertani broth *P. aeruginosa* was cultured in Nutrient broth containing 1% glycerol^42^. It was determined in preliminary experiments that Nutrient broth with 1% glycerol resulted in the highest levels of pyocyanin production by *P. aeruginosa* when compared to several other culture media (data not shown). All plates were incubated statically at 37 °C for 24 h for *C. violaceum* and for 48 h for *P. aeruginosa*. Total bacterial growth was estimated by determining the optical density of each culture at 600 nm before and after the incubation period and prior to pigment extraction.

Violacein from *C. violaceum* was quantified by transferring 1 ml from each plate well to a microfuge tube then pelleting the cells by centrifugation and discarding the supernatant. Dimethylsulfoxide (1 ml) was added to the cell pellet, which was resuspended by vigorous vortex mixing. Cells were pelleted by centrifugation again and the optical density of the supernatant (200 µl), which contained the violacein, was determined at 585 nm using dimethylsulfoxide as the spectrophotometer blank^43^.

Pyocyanin extraction was adapted from previously published methods^44,45^. Briefly, 1.5 ml was transferred from each plate well to a microfuge tube and cells were pelleted. The supernatant from each tube was collected, filter sterilized and then 0.6 ml of chloroform was added to 1 ml of each culture filtrate. Tubes were mixed thoroughly, then 0.4 ml of the bottom chloroform layer was transferred into a new microfuge tube. To this, 200 µl of 0.2 M hydrochloric acid was added, mixed thoroughly then centrifuged for 2 min at 10,000 rpm. From the resulting top layer, 200 µl was transferred to a 96-well plate and the optical density was determined at 520 nm using 0.2 M HCl as the spectrophotometer blank.

### Statistical analyses

Unless stated otherwise, all assays were repeated at least three times on separate occasions, and the mean and standard deviation was calculated. MICs with and without catalase were analysed by Student’s one-tailed paired t-tests. MICs from the hydrogen peroxide accumulation assay were analysed via Student’s two-tailed t-test, assuming equal variance. MICs with membrane modifiers were analysed via one-way analysis of variance (ANOVA), followed by Tukey’s multiple comparison post-hoc test, which reduces the risk of a type 1 error by using statistical hypothesis testing. Biofilm formation (crystal violet staining) data and accompanying total bacterial growth data, biofilm metabolic activity data (TTC metabolism) and pigment production data were analysed by two-way ANOVA followed by Tukey’s post-hoc test. Viable count data from the LCWPB method was analysed by one-way ANOVA followed by Tukey’s post-hoc test. Significance levels for all statistical analyses were set at 0.05.

## Results

### Physicochemical properties

Physical and chemical properties of the honeys are shown in Table 1, with Manuka honey being the darkest in colour, followed by the two Jarrah honeys. Both the multifloral and Manuka honeys produced negligible hydrogen peroxide, whereas overall, the highest amount was produced by Marri 1 at the 6 h time point.

**Table 1:** Physical and chemical parameters of honeys (mean values).

Honey	pH	Colour pre- and post-filtration (mAU)^a^		Phenolic content (mg GAE^b^ per 100 g)	Hydrogen Peroxide (µM) accumulated at each time point				
		Pre	Post		1 h	2 h	4 h	6 h	24 h
Jarrah 1	4.84	766	615	127.4	59	83	103	98	29
Jarrah 2	4.83	867	757	123.7	28	10	5	76	8
Marri 1	4.28	233	149	61.1	72	166	295	479	416
Marri 2	4.81	341	242	65.7	20	40	100	121	7
Manuka	4.00	1604	818	150.0	nd	nd	nd	nd	nd
Multifloral	4.24	431	209	72.0	nd	nd	nd	nd	nd

^a^Filtration with a 0.7 µm glass fibre filter; ^b^Gallic acid equivalent; nd not detected.

### Antibacterial activity

Honeys were screened for antibacterial activity against *S. aureus* ATCC 700699 and resulting mean zone sizes were 15.3 mm for Jarrah 1, 15.7 mm or Jarrah 2, 14.7 mm for Marri 1 and 15.5 mm for Marri 2. No zones were obtained for the multifloral honey and the Manuka honey, and trimethoprim (5 µg) resulted in a mean zone size of 26.3 mm. Results of the phenol equivalence assay showed phenol equivalence values of 34 for both Jarrah 1 and Marri 1, 18 for Jarrah 2 and 4.5 for Marri 2. Similar to the agar diffusion assay, no activity was detected for the Manuka honey or multifloral honeys by the phenol equivalence method.

MICs for the six honeys against four organisms are shown in Table 2. In addition, MICs for artificial honey were 40.7% for *S. aureus* ATCC 700699, 30.0% for *P. aeruginosa* ATCC BAA-47, 32.7% for *E. coli* NCTC 10538 and 37.3% for *E. faecalis* NCTC 775. Comparison of honeys showed that MICs were lowest for Jarrah 1, and whilst both Jarrah honeys were broadly similar in activity, the two Marri honeys differed in activity. The addition of catalase enzyme reduced antibacterial activity and increased MICs significantly (P value range 0.001–0.043) for all four organisms against both Jarrah and Marri honeys. The only exception was Marri 2 against *E. faecalis*, for which no significant change occurred. Little change in activity was observed for Manuka honey. The average increase in MIC for all four test organisms was 2.1% for Manuka, 9.2% for Marri 2, 15.0% for Marri 1, 15.4% for Jarrah 2 and 17.5% for Jarrah 1. Modal MICs of hydrogen peroxide alone were 128 µM for *S. aureus* ATCC 700699, 512 µM for *P. aeruginosa* ATCC BAA-47, 1024 µM for *E. coli* NCTC 10538 and 2048 µM for *E. faecalis* NCTC 775. When hydrogen peroxide was allowed to accumulate in honey prior to antibacterial testing, modest changes in activity were observed (Table 3). Marri 2, whilst producing the highest levels of hydrogen peroxide, was not able to be tested in this assay due to the low quantity of sample remaining. MICs of Jarrah 2 with and without the accumulation of hydrogen peroxide differed significantly (P = 0.025) against *S. aureus* ATCC 700699 only, whereas for Marri 2 MICs differed significantly for both *S. aureus* ATCC 700699 (P = 0.012) and *E. faecalis* NCTC 775 (P = 0.026). Not all changes in activity were statistically significant due to the size of the standard deviations. No significant differences were observed for manuka honey. The pH of Jarrah 2 solution was 4.61 after 1 h and 4.56 after 3 h. For Marri 2 the pH was 4.59 after 1 h and 4.49 after 3 h, and for Manuka the pH was 3.83 after 1 h and 3.77 after 3 h.

**Table 2:** MICs of honey (mean % w/v) in the presence and absence of catalase enzyme.

Honey	Organism	MIC	MIC + Catalase	Difference in MIC	P value^a^
Jarrah 1	*S. aureus* ATCC 700699	6.7	26.7	+20.0	**0.007**
	*E. faecalis* NCTC 775	13.3	31.3	+18.0	**0.002**
	*E. coli* ATCC 10538	13.3	27.3	+14.0	**0.003**
	*P. aeruginosa* ATCC BAA-47	10.0	28.0	+18.0	**—**
Jarrah 2	*S. aureus* ATCC 700699	8.7	27.3	+18.7	**0.008**
	*E. faecalis* NCTC 775	16.0	32.0	+16.0	**0.010**
	*E. coli* ATCC 10538	16.7	28.7	+12.0	**0.005**
	*P. aeruginosa* ATCC BAA-47	14.0	28.7	+14.7	**0.001**
Marri 1	*S. aureus* ATCC 700699	9.3	30.0	+20.7	**0.001**
	*E. faecalis* NCTC 775	21.3	32.0	+10.7	**0.043**
	*E. coli* ATCC 10538	17.3	31.3	+14.0	**0.022**
	*P. aeruginosa* ATCC BAA-47	12.0	26.7	+14.7	**0.001**
Marri 2	*S. aureus* ATCC 700699	16.0	30.7	+14.7	**0.034**
	*E. faecalis* NCTC 775	28.0	31.3	+3.3	NS
	*E. coli* ATCC 10538	22.0	30.0	+8.0	**0.010**
	*P. aeruginosa* ATCC BAA-47	18.0	28.7	+10.7	**0.024**
Manuka	*S. aureus* ATCC 700699	12.0	14.0	+2.0	NS
	*E. faecalis* NCTC 775	16.7	17.0	+0.3	—
	*E. coli* ATCC 10538	16.7	19.0	+2.3	—
	*P. aeruginosa* ATCC BAA-47	17.3	21.0	+3.7	NS
Multifloral	*S. aureus* ATCC 700699	32.0	Not done		
	*E. faecalis* NCTC 775	32.0	Not done		
	*E. coli* ATCC 10538	32.7	Not done		
	*P. aeruginosa* ATCC BAA-47	30.0	Not done		

^a^Data was analysed by Student’s one-tailed paired t-test, assuming equal variance. P values in bold indicate a significant difference (P < 0.05). NS: Not significant.

- No P value attainable due to absence of variance between experimental repeats.

**Table 3:** MICs of honey (mean % w/v) before and after the accumulation of hydrogen peroxide for 3 h.

Honey	Organism	Accumulation period		Difference in MIC	P value^a^
		1 h	3 h		
Jarrah 2	*S. aureus* ATCC 700699	8.7	4.0	−4.7	**0.025**
	*E. faecalis* NCTC 775	16.0	12.0	−4.0	0.158
	*E. coli* ATCC 10538	16.7	14.0	−2.7	0.374
	*P. aeruginosa* ATCC BAA-47	14.0	10.0	−4.0	0.116
Marri 2	*S. aureus* ATCC 700699	16.0	10.0	−6.0	**0.012**
	*E. faecalis* NCTC 775	28.0	24.0	−4.0	**0.026**
	*E. coli* ATCC 10538	22.0	22.7	+0.7	0.374
	*P. aeruginosa* ATCC BAA-47	18.0	18.0	0.0	1.000
Manuka	*S. aureus* ATCC 700699	12.0	10.0	−2.0	0.374
	*E. faecalis* NCTC 775	16.7	16.0	−0.7	0.374
	*E. coli* ATCC 10538	16.7	16.0	−0.7	0.374
	*P. aeruginosa* ATCC BAA-47	17.3	16.0	−1.3	0.374

^a^Data was analysed by Student’s two-tailed t-test, assuming equal variance. P values in bold indicate a significant difference (P < 0.05).

The effects of membrane-modifying compounds on susceptibility to honey showed a range of effects (Table 4). The mean change in MIC for all three honeys and four organisms in the presence of PMBN was 1.02%, indicating a relatively small overall effect. Similarly, the mean change in MIC in the presence of CCCP was relatively small at 3.25%. The largest overall effect was seen with EDTA, with a mean change in MIC of 5.8%. However, consistent trends were not seen across organisms or honeys. For example, inclusion of PMBN resulted in significantly altered MICs for Manuka honey only, against the two *P. aeruginosa* strains (P values 0.005–0.012), but not the two *E. coli* strains. Similarly, whilst the presence of EDTA significantly altered Manuka honey MICs for all organisms (P value range 0.001–0.030), EDTA resulted in significantly altered MICs for Marri 1 only, against the two *P. aeruginosa* strains (P values 0.029–0.032), and for Jarrah 1, MICs with and without EDTA only differed significantly for one *E. coli* strain (P = 0.003). Analysis of MICs obtained in the presence of EDTA using an alternative statistical test (one-tailed Students t test assuming equal variance) showed than MICs obtained with EDTA were significantly lower (P value range 0.001 to 0.048) than those obtained without EDTA, for all honeys and organisms except for both *P. aeruginosa* strains with Jarrah 1 and PAO1 with Manuka (P > 0.05).

**Table 4:** MICs of honey (mean % w/v) or novobiocin (mode; µg/ml) against Gram negative bacteria in the presence and absence of membrane modifying compounds.

Honey / Antibiotic	Organism	MICs with or without treatment			
		None	PMBN^a^	EDTA	CCCP
Jarrah 1	*E. coli* ATCC 10538	13.3	12.5	**7.5**	10.8
	*E. coli* ATCC 25922	12.5	13.3	5.8	6.7
	*P. aeruginosa* ATCC BAA-47	10.0	9.2	8.8	7.5
	*P. aeruginosa* ATCC 27853	10.0	10.0	10.0	10.0
Marri 1	*E. coli* ATCC 10538	15.2	15.0	5.0	10.8
	*E. coli* ATCC 25922	15.3	16.7	10.0	**6.7**
	*P. aeruginosa* ATCC BAA-47	13.0	10.8	**7.5**	12.5
	*P. aeruginosa* ATCC 27853	13.0	12.5	**8.8**	13.3
Manuka	*E. coli* ATCC 10538	15.8	12.5	**3.3**	10.8
	*E. coli* ATCC 25922	13.3	14.2	**4.2**	8.3
	*P. aeruginosa* ATCC BAA-47	15.0	**12.5**	**12.5**	13.3
	*P. aeruginosa* ATCC 27853	18.3	**13.3**	**11.7**	**15.0**
Novobiocin	*E. coli* ATCC 10538	128	<8	**—**	**—**
	*E. coli* ATCC 25922	64	<8	**—**	**—**
	*P. aeruginosa* ATCC BAA-47	>256	<8	**—**	**—**
	*P. aeruginosa* ATCC 27853	128	<8	**—**	**—**

Values in bold indicate a significant difference between treatment and the untreated control (without membrane treatment), determined by one-way ANOVA followed by Tukey’s post-hoc test.

^a^Final concentrations were as follows; PMBN: 10 μg/mL for both *E. coli* strains, 0.0625 μg/mL for *P. aeruginosa* ATCC BAA-47, and 0.3125 μg/mL for *P. aeruginosa* ATCC 27853; EDTA: 0.625 mM for both *P. aeruginosa* strains, 2.5 mM for *E. coli* NCTC 10538, and 5 mM for *E. coli* ATCC 25922; CCCP: 100 μM for both *E. coli* strains and 200 μM for both *P. aeruginosa* strains.

### Biofilm formation

**Figure 1:**
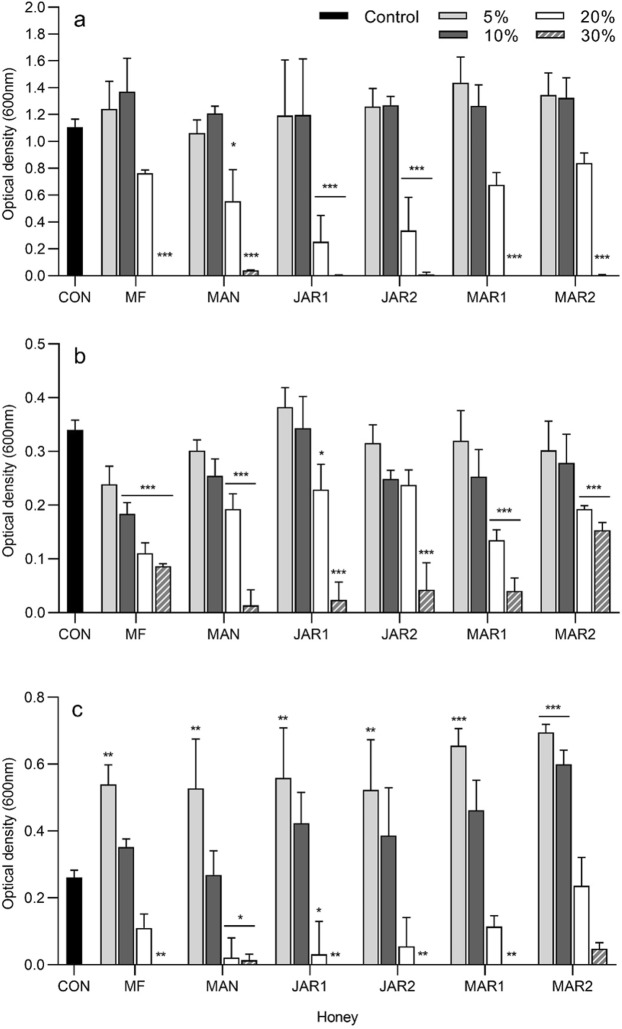
Bacterial growth after 24 h in the presence of several concentrations of honey, determined by optical density at 600 nm (mean and standard deviation). (**a**) *Pseudomonas aeruginosa* ATCC BAA-47 (**b**) *Enterococcus faecalis* NCTC 775 and **(c)** *Staphylococcus aureus* ATCC 700699. Data were analysed by 2-way ANOVA and Tukey’s multiple comparisons post-hoc test. Asterisks indicate significant differences compared to the untreated control (*P < 0.05; **P < 0.01; ***P < 0.001). CON untreated control; MF multifloral; MAN Manuka; JAR1 Jarrah 1; JAR2 Jarrah 2; MAR1 Marri 1; MAR2 Marri 2.

**Figure 2:**
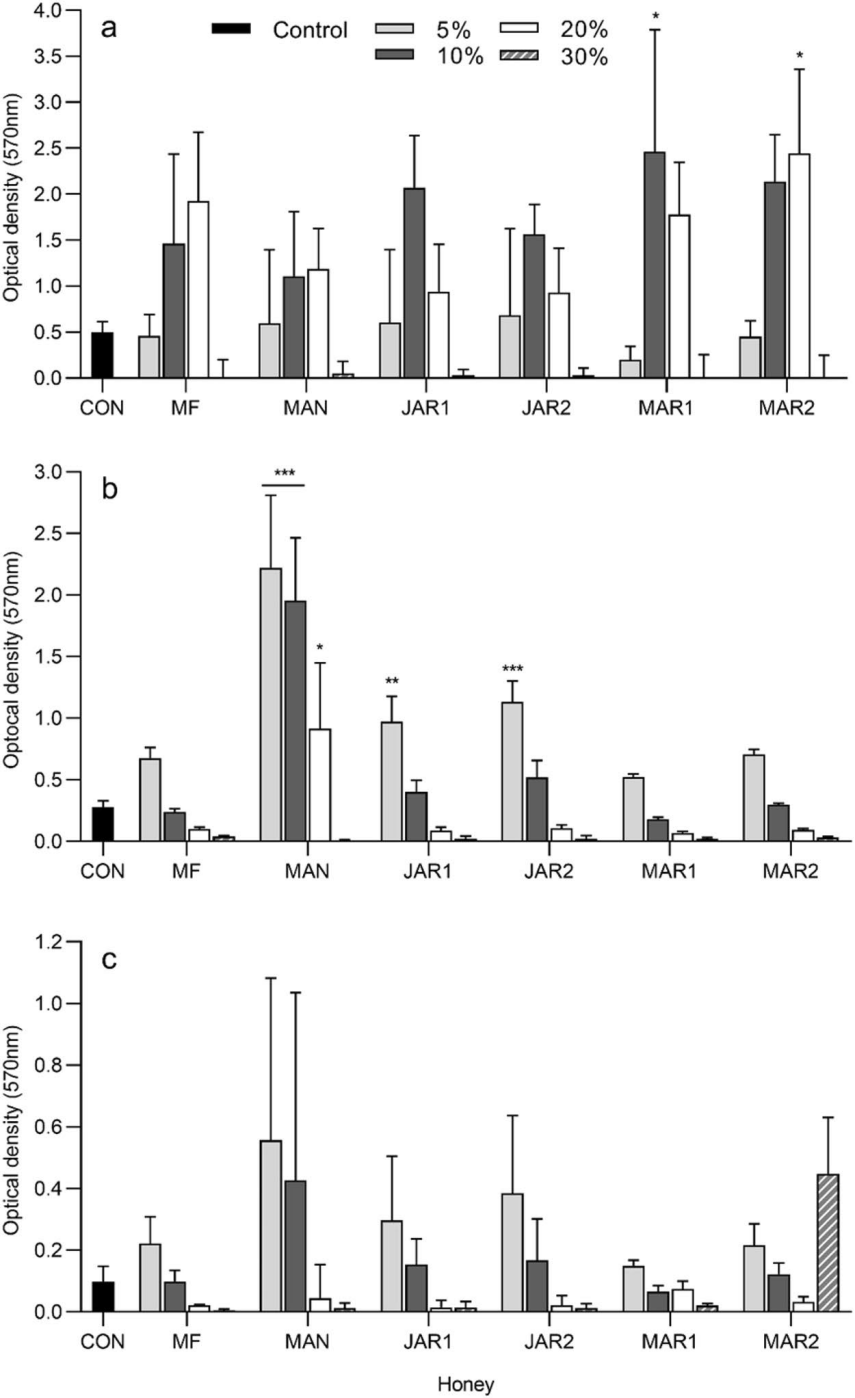
Biofilm formation after 24 h in the presence of several concentrations of honey, determined by crystal violet staining (mean and standard deviation). (**a**) *Pseudomonas aeruginosa* ATCC BAA-47 (**b**) *Enterococcus faecalis* NCTC 775 and (**c**) *Staphylococcus aureus* ATCC 700699. Data were analysed by 2-way ANOVA and Tukey’s multiple comparisons post-hoc test. Asterisks indicate significant differences compared to the control (*P < 0.05; **P < 0.01; ***P < 0.001). CON untreated control; MF multifloral; MAN Manuka; JAR1 Jarrah 1; JAR2 Jarrah 2; MAR1 Marri 1; MAR2 Marri 2.

Analysis of total bacterial growth (Fig. 1) by 2-way ANOVA showed that both the type and concentration of honey represented significant sources of variation (P ≤ 0.022) for all three organisms. Analysis of biofilm formation by 2-way ANOVA (Fig. 2) showed that concentration represented a significant source of variation (P ≤ 0.001) for all three organisms whereas honey type varied significantly for *E. faecalis* only.

Bacterial growth in the presence of 30% honey was virtually absent, or greatly reduced relative to the untreated control. Correspondingly, the formation of biofilm was largely absent in the presence of 30% honey. The exception was honey Marri 2 at 30%, which appeared to have considerable biofilm formation despite growth being greatly reduced at this concentration. However, in this particular instance neither the biofilm formation (P = 0.74) nor total growth (P = 0.097) differed significantly from the untreated control. At 20% honey, growth of *E. faecalis* was significantly reduced relative to the untreated control for all honeys except Jarrah 2 (P = 0.068), whereas for *S. aureus* growth was only significantly reduced by Manuka and Jarrah 1, and for *P. aeruginosa* was significantly reduced only by Manuka (P = 0.026), Jarrah 1 (P < 0.0001) and Jarrah 2 (P < 0.0001). Amounts of biofilm formed in the presence of 20% honey differed significantly from the untreated control in two instances only; for *P. aeruginosa* in the presence of 20% Marri 2 honey and *E. faecalis* in the presence of 20% Manuka honey. In both instances biofilm formation was substantially increased compared to the untreated control.

For *E. faecalis*, growth in the presence of 10% and 5% honey was for the most part similar to the untreated control and did not differ significantly, whereas biofilm was enhanced relative to the untreated biofilm control. Significant differences in *E. faecalis* biofilm compared to the control were found in the presence of 5 and 10% Manuka honey, and 5% for both Jarrah honeys. Similarly, growth for *P. aeruginosa* treated with 5% or 10% honey was largely similar to the untreated control and did not differ significantly and whilst biofilm formation appeared to be increased, this was only statistically significant for 10% Marri 1 honey (P = 0.011). *S. aureus* showed a slightly different trend, with growth significantly enhanced in the presence of all honeys at 5% (P ≤ 0.01), and also for 10% Marri 2 honey (P = 0.0001). Whilst biofilm appeared to be increased in line with the enhanced growth, biofilm formation did not differ significantly from the control for any honey treatment. This is likely due to the relative size of the standard deviation, which corresponds to the degree of biological variability frequently experienced with this particular microtitre biofilm assay. Significant differences were seen between biofilms formed in the presence of different honeys, however these significant differences were at dissimilar honey concentrations (for example 10% of one honey differed from another honey at 20%) and as such are likely to be related to concentration alone, rather than to specific differences in activity between honeys.

### Effect of honey on production of bacterial pigments

**Figure 3:**
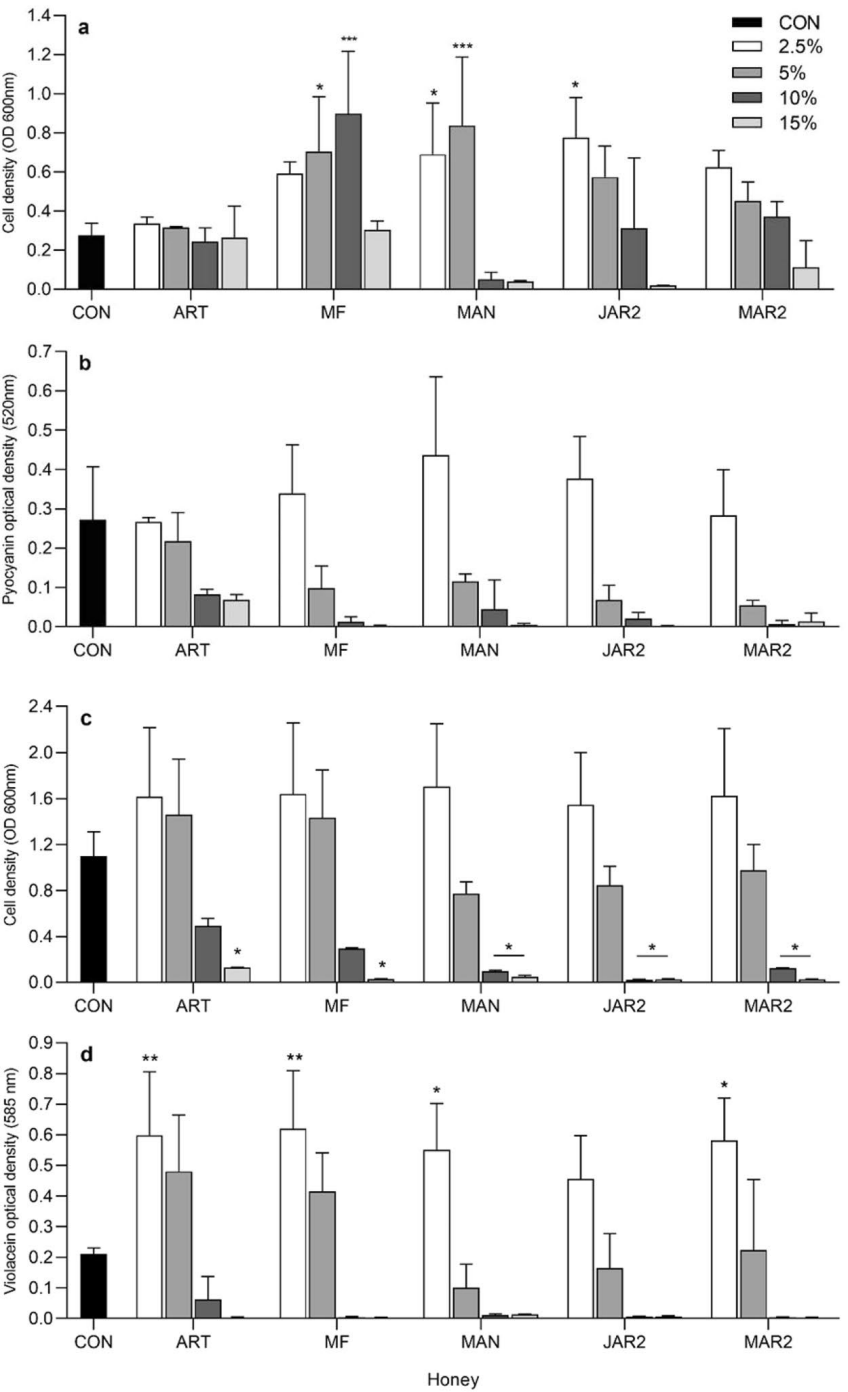
Bacterial growth and pigment production in the presence of several concentrations of honey (mean and standard deviation). Growth (**a**) and pyocyanin production (**b**) for *P. aeruginosa* ATCC 27853. Growth (**c**) and violacein production (**d**) for *C. violaceum* ATCC 12744. Data were analysed by 2-way ANOVA and Tukey’s multiple comparisons post-hoc test. Asterisks indicate significant differences compared to the control (*P < 0.05; **P < 0.01; ***P < 0.001). CON untreated control; ART artificial; MF multifloral; MAN Manuka; JAR2 Jarrah 2; MAR2 Marri 2.

For *P. aeruginosa*, both the type and concentration of honey significantly affected bacterial growth (P < 0.0001), whereas only the concentration significantly affected pyocyanin levels (P < 0.0001) (Fig. 3). Growth differed significantly from the untreated control at 2.5% and 5% Manuka honey (P ≤ 0.03), 5 and 10% multifloral honey (P ≤ 0.02) and 2.5% Jarrah 2 honey (P = 0.002). No significant differences were found for pyocanin levels obtained for the different treatments by Tukey’s post-hoc test. For *C. violaceum*, analysis by 2-way ANOVA showed that honey concentration significantly affected both growth (P < 0.0001) and violacein (P < 0.0001) results, whereas type of honey was a significant factor for violacein levels (P = 0.037), and not for bacterial growth. Post-hoc tests showed that growth differed significantly from the control at 15% for all honeys (P value range 0.014–0.044), and at 10% for Manuka (P = 0.031), Jarrah 2 (P = 0.013) and Marri 2 (P = 0.042). For violacein, levels differed significantly from the untreated control at the concentration of 2.5% only (P value range 0.003–0.035), for all honeys except Jarrah 2, which did not differ significantly (P = 0.41).

### Effects of honey on pre-formed biofilms

**Figure 4:**
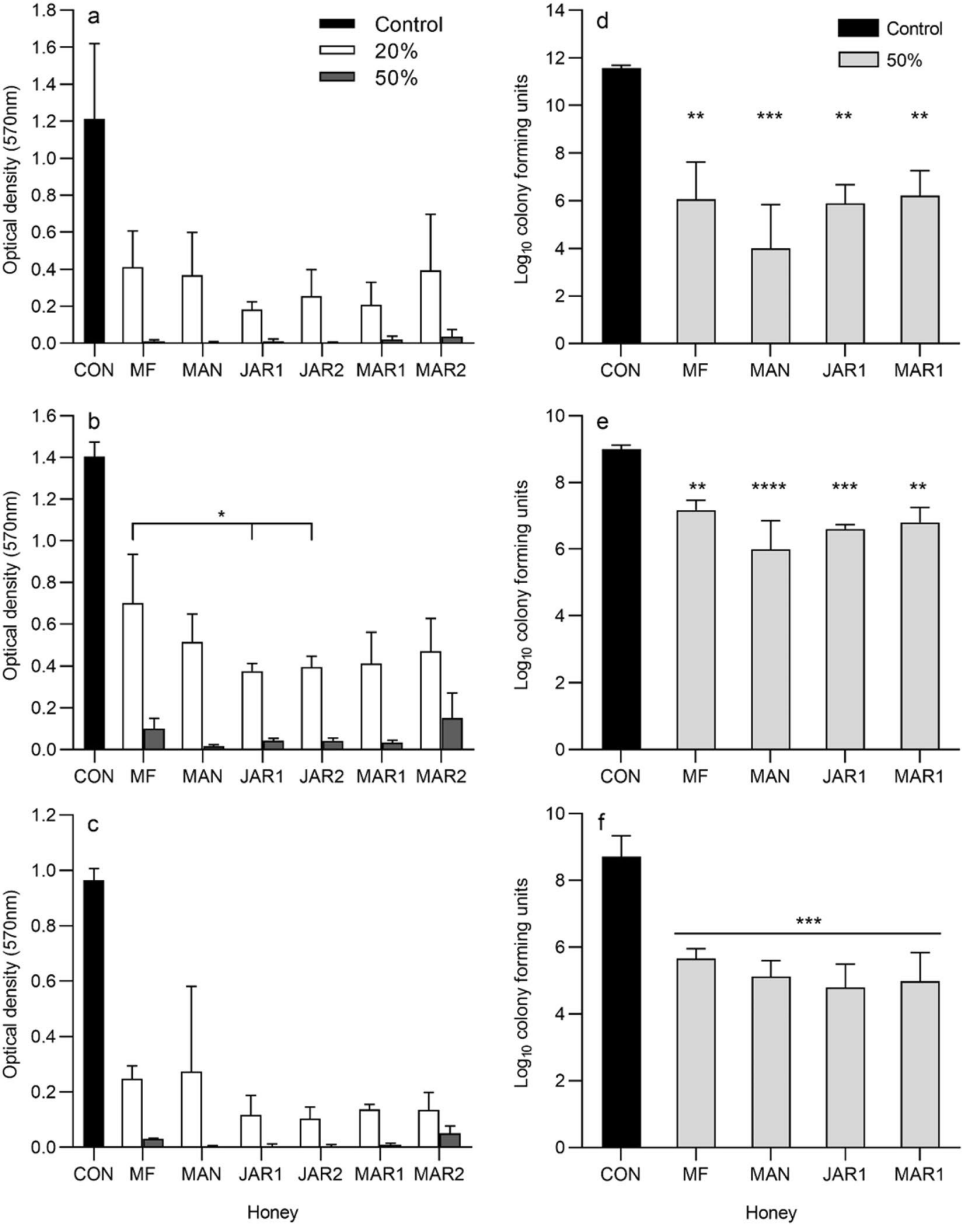
Metabolic activity and viability of biofilms after treatment with honey (mean and standard deviation). Metabolic activity was determined by TTC metabolism and viability was determined by viable counts. Metabolic activity of (**a**) *Pseudomonas aeruginosa* BAA-47 (**b**) *Enterococcus faecalis* NCTC 775 and (**c**) *Staphylococcus aureus* ATCC 700699, and viable counts of (**d**) *Pseudomonas aeruginosa* BAA-47 (**e**) *Enterococcus faecalis* NCTC 775 and (**f**) *Staphylococcus aureus* ATCC 700699 recovered from multispecies biofilms. Metabolic activity data were analysed by 2-way ANOVA and Tukey’s multiple comparisons post-hoc test. All honey-treated biofilms (**a**–**c**) differed significantly from the untreated control (significance not shown on graphs). Viable count data (**d**,**e**,**f**) was analysed by one-way ANOVA followed by Tukey’s post-hoc test and all honey-treated biofilms differed significantly from the untreated control. Asterisks indicate significant differences compared to the control (**d**–**f**), or between treatments (**b**) (*P < 0.05; **P < 0.01; ***P < 0.001). CON untreated control; MF multifloral; MAN Manuka; JAR1 Jarrah 1; JAR2 Jarrah 2; MAR1 Marri 1; MAR2 Marri 2.

Analysis of data quantifying the metabolic activity of pre-formed biofilms subsequently treated with either 20% or 50% honey (Fig. 4) by 2-way ANOVA showed that for all three test organisms, the concentration of honey represented a significant source of variation (P < 0.0001) whereas the type of honey did not (P value range 0.064–0.97). Post-hoc analysis showed that values obtained for all honey-treated biofilms differed significantly from the untreated control, for all three organisms. For *P. aeruginosa*, no significant differences between honeys were observed by post-hoc analysis. For *S. aureus*, post-hoc analyses showed that results from treatment with 20% Manuka differed significantly from 50% Manuka, Jarrah 1, Jarrah 2 and Marri 1 only. For *E. faecalis*, widespread significant differences between honey treatments were seen. For example, for each individual honey, the results obtained after treatment with 20% differed significantly from results obtained for that same honey at 50%. Significant differences were also observed between different honeys, but only when comparing 20% of one honey to 50% of another honey. Comparison of all honeys at the same concentration of 20% showed no significant differences between honeys against both *S. aureus* and *P. aeruginosa*. For *E. faecalis*, however, multifloral honey at 20% differed significantly from both 20% Jarrah 1 (P = 0.021) and 20% Jarrah 2 (P = 0.041). Analysis of results obtained for all biofilms treated with 50% honey showed no significant differences.

When honey was applied to multispecies biofilms formed within a simulated chronic wound environment (Fig. 4), significant effects on the viability of each organism were observed (P ≤ 0.0002) Post-hoc analysis showed similar trends for all three organisms, whereby results for all honey treatments differed significantly from the untreated control, but honeys did not differ significantly from each other.

## Discussion

Western Australia has a rich and diverse range of endemic flora^46^, from which bees collect nectar and pollen. Bees transform the nectar into honey, which in some instances may be considered to be a monofloral honey, depending on the relative contribution of each specific source of nectar and pollen. A range of health benefits have historically been ascribed to various monofloral honeys, however, there are very few scientific publications that describe the medicinal properties (including antibacterial activity) or therapeutic benefits of honeys derived from Western Australian flora^7,8,47,48^.

In the current study, all four Western Australian honeys showed antibacterial activity, and had notably higher activity than both the multifloral and artificial honeys, and in some instances were lower than the manuka honey. Marri 2 was slightly less active that the remaining three WA honeys, for as yet undetermined reasons. Comparison to a previous study that also evaluated Jarrah, Marri, multifloral and *Leptospermum* honeys showed that MICs were broadly similar^8^. However, an important difference is that in the current study, honeys were tested in 2% increments rather than doubling dilutions as described by Roshan *et al*., (2017), which allowed for substantially greater discrimination of activity. Comparison to a selection of honeys from different geographical areas showed that MICs for Jarrah and Marri were similar to values published for monofloral honeys (and obtained using methodology similar to the current study), such as European honeydew from Slovakia (MICs of 12.5%)^49^ chestnut and pine honeys from Greece (MICs of 3.1–6.2%)^50^ heather and buckwheat honeys from Poland (MICs of 3.1–25%)^51^ and blueberry and buckwheat honeys from Canada (MICs of 4–16%)^16^, but were generally lower than those reported for European Hawthorn from Slovakia (MIC > 25%)^49^ and Rapeseed honey from Greece (MIC of ≥25%)^49,50^. MICs for WA honeys were also similar to those previously obtained for manuka honeys^16,26,49,51,52^, but lower MICs have been published previously for honeys such as buckwheat^19^, heather^20^ and chestnut^21^. Comparison of the relative susceptibility of each test organism showed that *S. aureus* was the most susceptible to all four monofloral honeys and manuka, whilst the remaining three test species were similar to each other in susceptibility. Whilst some researchers have observed a similar trend in organism-specific susceptibility^8,50^, others have found no such trend^47^, suggesting that patterns in activity are likely to be both fairly complex and honey-dependent. Phenol equivalence or “total activity” values were largely in agreement with those published in two previous studies^7,8^

Hydrogen peroxide is one factor that has been shown to contribute to the antibacterial activity of honey^52–54^, including several from Western Australia^7,8^. Hydrogen peroxide is produced in honey when the enzyme glucose oxidase reacts with glucose and water, to produce gluconic acid and hydrogen peroxide. Results from the current study confirmed that the addition of catalase enzyme to degrade hydrogen peroxide significantly reduced the activity of both Jarrah and both Marri honeys. The activity remaining after removal of hydrogen peroxide was similar to that of multifloral honey. However, it remains possible that additional antimicrobial compounds or factors are present at low concentrations in Jarrah and Marri honeys and further studies are required. No significant change in activity was observed for the Manuka honey after the addition of catalase. This was expected given that hydrogen peroxide is known not to accumulate in Manuka honeys, possibly due to an interaction between methylglyoxal and the glucose oxidase enzyme^55^. The role of hydrogen peroxide in antibacterial activity was further demonstrated in the current study by showing that activity increased significantly when hydrogen peroxide was allowed to accumulate in honey solutions for 3 hours prior to testing. Whilst the kinetics of hydrogen peroxide accumulation in honey have been studied previously,^10,55,56^ to the best of our knowledge this is the first time that associated changes in antibacterial activity have been reported. The pH of each solution decreased by a relatively small amount over the 3 hour accumulation period, and it remains unclear whether this minor pH shift may have contributed to the observed additional antibacterial activity.

Several interesting observations arose from tests examining the contribution of hydrogen peroxide to antibacterial activity. Firstly, the levels of hydrogen peroxide generated in honey solutions were substantially lower than the MICs of hydrogen peroxide alone, with the possible exception of *S. aureus*, for which the hydrogen peroxide MIC was relatively low. This suggests that hydrogen peroxide levels in honey solutions may be too low to have any direct antibacterial activity, and instead may be acting in an additive or synergistic manner with other honey components such as the relatively low pH environment, organic acids, plant-derived components or osmotic activity. Secondly, the honeys with the greatest changes in antibacterial activity after the addition of catalase were not the same as those that produced the highest levels of hydrogen peroxide. For example, both Jarrah honeys and Marri 2 showed similar decreases in antibacterial activity, yet produced comparatively different levels of hydrogen peroxide. More specifically, Jarrah 2 accumulated relatively inconsequential levels of hydrogen peroxide over the 24 h test period, yet negating the hydrogen peroxide accumulation by adding catalase enzyme resulted in a relative large decrease in antibacterial activity. Furthermore, allowing hydrogen peroxide to accumulate in solutions of honey Jarrah 2 prior to testing for antibacterial activity resulted in a significant increase in antibacterial activity, although only against *S. aureus*. Although the method used in the current study to quantify hydrogen peroxide has been used previously in honey research^17^, it remains possible that the results generated do not accurately reflect true levels of hydrogen peroxide. This could explain the apparent disconnect between hydrogen peroxide results and antibacterial activity and warrants further investigation. As a whole, these antibacterial activity data indicate that even relatively modest levels of hydrogen peroxide may contribute substantially to antibacterial activity, and that the contribution of hydrogen peroxide to overall activity may be more complex than previously thought. Similar conclusions have been reached by other researchers that have observed complex interactions between hydrogen peroxide and other honey components^9,19,53,57^. The contribution of bee defensin (an antibacterial peptide) to the antibacterial activity of honey has also been noted previously^12,13,27,58^, but has not been investigated in Western Australian honeys and is therefore of considerable interest for future studies.

The observation that *S. aureus* was generally the most susceptible of the test organisms to honeys prompted the investigation of factors that may contribute to differences in susceptibility between organisms. In particular, the Gram negative outer membrane is an obvious difference between Gram positive and Gram negative bacteria. It is an effective permeability barrier to large hydrophilic compounds and to hydrophobic compounds, whereas small hydrophilic compounds can gain entry to the cell via outer membrane porins^59^. Examination of the role of the outer membrane may indicate whether it is able to exclude antibacterial compounds present in honey, thereby reducing the antibacterial effects. When PMBN was included in the MIC assays, no consistent trend was observed and only minor differences in MICs of honey were observed, whilst substantial effects were seen for novobiocin. Whilst these data did not indicate that the outer membrane has a significant role in susceptibility to honey, tests with EDTA showed a potential effect. Notably, MICs of Manuka honey with EDTA were significantly decreased for all four test organisms, providing the most compelling evidence that the outer membrane has a role in susceptibility to honey. EDTA is a known chelator, removing calcium and magnesium cations from the Gram negative outer membrane and destabilising it^59^. It is possible that this destabilisation allowed the entry of large antibacterial molecules into the cell, or enhanced the antimicrobial effects of other antibacterial factors, such as osmotic stress. Further studies are required to elucidate the exact role of the outer membrane in susceptibility to honey. Lastly, the addition of CCCP to depolarise the cytoplasmic membrane did not result in large differences or show a consistent overall trend, leading to the presumption that cytoplasmic membrane polarity does not play a substantial role in susceptibility to honey. It was also interesting to note that of the eleven instances where MICs were significantly altered in the presence of membrane modifiers, seven were for Manuka honey. This suggests that the effect may be more pronounced for, or more specific to Manuka honey. This in turn suggests that destabilisation of the outer membrane may impact on the capacity for Manuka-specific antimicrobial compounds to enter the bacterial cell. Methylglyoxal is unlikely to be one such compound as it is not a particularly large molecule, and is reported to be freely soluble across membranes^60^. Other unique compounds found in Manuka honey, such as the glycoside leptosperin^61^ may be candidates. Several studies have examined the effects of honey on bacterial membranes^62,63^ but to the best of our knowledge none have examined the effect of membrane destabilisation on susceptibility. Data from the current study indicate that the Gram negative outer membrane may play a role in reducing the antibacterial effects of Western Australian honeys. However, the specific role requires further investigation.

Observations from the current study of differences in susceptibility between bacterial species also prompted the question of whether this trend would also be seen in a broader context, and in particular in biofilm formation. This is highly relevant as one of the major clinical applications for honey is in the treatment of chronic, infected wounds^52,56^. Microbial biofilms typically contain multiple microbial species and can be difficult to eradicate, which contributes to the chronicity of these wounds^29,30^. Data from the current study showed that both biofilm formation and bacterial growth were significantly altered in the presence of honey. For the most part, different honeys were indistinguishable in activity when assessed using the biofilm formation assay. A closer evaluation of results showed that the effects on biofilm formation could be grouped into four broad patterns or trends, which were broadly related to honey concentration.

The first trend was where both bacterial growth and biofilm formation were severely restricted, and this occurred at the higher concentration of 30% honey, for most organisms and most honeys. Similar inhibition of biofilm formation at relatively high concentrations has been demonstrated previously for a range of honeys types, including manuka^25,64^ and Norwegian forest honey^65^. This global growth inhibition is a direct result of the antibacterial activity of the honeys and is likely due to effects including osmotic stress, coupled with low pH and the presence of hydrogen peroxide. The second trend was where biofilm formation was enhanced, but appeared to be a direct result of enhanced bacterial growth. For example, this was observed for *S. aureus* treated with several different honeys at 2.5%. This enhancement or stimulation of biofilm formation has been observed in previous studies with honeys including Surgihoney^18^ and medihoney (manuka)^25,26^, and may be due to the stimulatory effect of the sugars present within the honey on bacterial growth. Thirdly, in some instances, biofilm formation was enhanced in the absence of increased overall growth, suggesting a specific upregulation of biofilm formation. Examples of this are *P. aeruginosa* treated with 10% and 20% multifloral honey and *E. faecalis* treated with 5% Manuka honey. A diverse range of compounds, including antibiotics, have been shown previously to stimulate biofilm formation^66,67^ indicating that this effect is not unique to honey. In addition, hydrogen peroxide has been shown in several studies to also stimulate biofilm formation; for example in *Acinetobacter oleivorans*^68^ by stimulating the production of exopolysaccharide and in *P. aeruginosa* by stimulating alginate production^69^. Lastly, there were a few instances where biofilm was reduced relative to growth for that same honey treatment. Examples include *S. aureus* treated with Marri 1 and Marri 2 at 10%, where growth was enhanced but biofilm was not. This may indicate a specific anti-biofilm effect and further studies are warranted to investigate potential underlying mechanisms. However, this study also highlighted an issue with the biofilm microtitre method that has been observed previously by others^70^, whereby in some instances relatively large variation occurred between individual test repeats. This was most pronounced for *S. aureus*, but also occurred with the remaining test organisms.

Following on from biofilm formation studies, experiments were conducted to investigate whether changes in biofilm formation may be related to alterations in quorum sensing. Quorum sensing is the cell to cell communication system used to coordinate and regulate the behaviour of cell populations, including the production of virulence factors and formation of biofilm. It is achieved by the production, and detection, of signalling molecules known as autoinducers^44^. Quorum sensing was investigated indirectly, using two widely studied Gram negative strains that produce pigments, the production of which is regulated by quorum sensing. Similar to biofilm studies, honey showed a largely dose-dependent effect on growth, including both stimulation and inhibition. For *C. violaceum*, trends in pigment production appeared to mirror general bacterial growth patterns, whereas for *P. aeruginosa* there were several instances where growth was significantly enhanced but pyocyanin production was relatively diminished, suggesting that a specific inhibition of quorum sensing may be occurring under those specific conditions. Several studies have found that phenolic compounds identified in some *Eucalyptus* honeys^47,71^ inhibit either virulence or quorum sensing. Examples include the flavonoid myricetin, which has been shown to inhibit virulence in *S. aureus* by downregulating the *saeR* global regulator^72^, and phenyllactic acid^73^ which inhibited virulence and quorum sensing by interfering with *P. aeruginosa* autoinducer binding receptors^73^. In contrast, a study demonstrating the inhibition of pyocyanin production and downregulation of the expression of quorum sensing genes in *P. aeruginosa* after treatment with Manuka honey attributed the effects to the sugar content, and in particular glucose^74^. Further work is therefore warranted to elucidate potential mechanisms by which inhibition of pyocyanin production may be occurring, and the components responsible for the effect.

Overall, data from biofilm formation and pigment production studies broadly indicate that inhibition of both occurs via a non-specific mechanism and is a result of global growth inhibition. However, it remains possible that the specific inhibition of quorum sensing may be occurring under particular conditions. Stimulation of biofilm formation by honey occurred at low concentrations, which is unlikely to be clinically relevant given that honey is typically applied undiluted in a clinical setting^75^, and enhancement of growth or biofilm is therefore unlikely to occur *in vivo* as long as concentrations of honey remain relatively high.

Honey also affected preformed biofilms, by reducing the metabolic activity of single-species biofilms, and reducing viability in multispecies biofilms. Relatively high concentrations (20% and 50%) of honey were evaluated as it is well known that established biofilms are typically less susceptible to antimicrobial agents compared to planktonic cells, and sub-inhibitory concentrations of honey have been shown to actually increase the biomass of existing biofilms^26^. Decreases in the metabolic activity of biofilms occurred even after the application of 20% (w/v) honey, which is noteworthy given that 20% honey was not always sufficient to prevent growth or the development of biofilm in this study. Results obtained for different types of honeys in the metabolic activity assay did not differ significantly for *S. aureus* or *P. aeruginosa*, suggesting that the honeys had indistinguishable activity under these conditions. In contrast, results for *E. faecalis* treated with the two Jarrah honeys at 20% differed significantly from multifloral honey at 20%, suggesting that specific components in the Jarrah honeys, such as hydrogen peroxide or plant-derived components, provided additional activity. Application of 50% (w/v) honey almost completely abolished metabolic activity in single-species biofilms, whereas examination of cell viability in the multispecies biofilm model showed significant but relatively modest reductions after the application of 50% honey. Manuka honey has been shown previously to reduce the viability of cells within single-species biofilms of *Enterobacter cloacae* and *Proteus mirabilis*^49^ and *S. aureus*^26^. The effects of honeys on established, multi-species biofilm has previously been investigated by Sojka *et al*. (2016), who found that both manuka and honeydew applied topically at 100% effectively reduced viability of several bacterial species within established biofilms, with the exception of *E. faecalis*^27^. Whilst data from the current study provide proof-of-concept that honeys can exert a bactericidal effect in a simulated wound environment, they also indicate that only a small proportion of bacterial cells may actually be dead as a result of honey treatment, and that any remaining viable cells could recover after the cessation of honey treatment. Multiple applications of honey may therefore be required to further reduce the viability of cells within an established biofilm. Data from the current study demonstrate that several honeys are able to prevent the formation of biofilm, most likely via a non-specific mechanism such as the inhibition of bacterial growth. In addition, honey was shown to reduce the metabolic activity of formed biofilms, and also reduce numbers of viable cells within a multi-species biofilm. These data provide a significant contribution to the body of pre-clinical evidence in support of the use of honey for wound treatment.

The therapeutic efficacy of honey has been evaluated for the treatment of a range of medical conditions (both with and without a microbial component), including radiation-induced mucositis^76^, venous leg ulcers^75^, prevention of surgical site infection^77^ and nocturnal cough in children^78^. Several of these studies were conducted with Manuka honey, which is one of only a few honeys worldwide that have been developed into registered therapeutic products. Other registered therapeutic honey products include Revamil^79^ and Surgihoney^10,25^, both of which have substantial hydrogen peroxide activity. Although differences in antibacterial activity between honeys can be quantified *in vitro*, to date there is little evidence to indicate whether these differences translate into significantly different outcomes in a clinical setting. Furthermore, results from several experiments described in the current study indicate that honeys do not differ significantly from each other in activity against biofilms. This supports the notion that although statistical differences in activity can be measured using MIC assays, these differences may not in fact be biologically or clinically relevant, and may not actually translate into better (or worse) clinical efficacy. This highlights some of the limitations of the interpretation of statistical analyses, and the importance  of also interpreting results in a biological or clinical context. In addition, a limitation of this study was that a relatively small number of honey samples were investigated, meaning that the study was likely underpowered to accurately detect significant differences between honeys.

In conclusion, this study has demonstrated that Western Australian honeys are comparable to manuka honey with regards to antibacterial activity and effects on bacterial biofilms. A range of further studies are warranted, including further exploration of the role of the Gram negative outer membrane, investigation of bee defensin as a potential antibacterial component of honey, and clinical studies to determine whether the therapeutic treatment with different honeys with relatively higher or lower antibacterial activity correlates with differences in therapeutic outcomes.
